# Alzheimer’s Disease and Diabetes: Role of Diet, Microbiota and Inflammation in Preclinical Models

**DOI:** 10.3390/biom11020262

**Published:** 2021-02-10

**Authors:** Maria Jose Carranza-Naval, Maria Vargas-Soria, Carmen Hierro-Bujalance, Gloria Baena-Nieto, Monica Garcia-Alloza, Carmen Infante-Garcia, Angel del Marco

**Affiliations:** 1 Division of Physiology, School of Medicine, Universidad de Cadiz, 11003 Cadiz, Spain; mariajosecarranza@uca.es (M.J.C.-N.); mariavargas@uca.es (M.V.-S.); carmenhierro@uca.es (C.H.-B.); monica.garcia@uca.es (M.G.-A.); 2 Instituto de Investigacion e Innovacion en Ciencias Biomedicas de la Provincia de Cadiz (INIBICA), 11009 Cadiz, Spain; gbaenanieto@gmail.com; 3 Salus Infirmorum, Universidad de Cadiz, 11005 Cadiz, Spain; 4 Department of Endocrinology, Jerez Hospital, Jerez de la Frontera, 11407 Cadiz, Spain

**Keywords:** Alzheimer’s disease, type 2 diabetes mellitus, microbiota, diet, microglia, inflammation

## Abstract

Alzheimer’s disease (AD) is the most common cause of dementia. Epidemiological studies show the association between AD and type 2 diabetes (T2DM), although the mechanisms are not fully understood. Dietary habits and lifestyle, that are risk factors in both diseases, strongly modulate gut microbiota composition. Also, the brain-gut axis plays a relevant role in AD, diabetes and inflammation, through products of bacterial metabolism, like short-chain fatty acids. We provide a comprehensive review of current literature on the relation between dysbiosis, altered inflammatory cytokines profile and microglia in preclinical models of AD, T2DM and models that reproduce both diseases as commonly observed in the clinic. Increased proinflammatory cytokines, such as IL-1β and TNF-α, are widely detected. Microbiome analysis shows alterations in *Actinobacteria*, *Bacteroidetes* or *Firmicutes* phyla, among others. Altered α- and β-diversity is observed in mice depending on genotype, gender and age; therefore, alterations in bacteria taxa highly depend on the models and approaches. We also review the use of pre- and probiotic supplements, that by favoring a healthy microbiome ameliorate AD and T2DM pathologies. Whereas extensive studies have been carried out, further research would be necessary to fully understand the relation between diet, microbiome and inflammation in AD and T2DM.

## 1. Alzheimer’s Disease

Growing life expectancy is secondarily contributing to a significant rise of pathologies associated with aging. Among them, dementias are specially relevant due to their prevalence, economical costs and societal burden [1]. Alzheimer’s disease (AD) is the most prevalent form of dementia, which may contribute to 60–70% of total cases [2], followed by vascular dementia. AD neuropathological features include: amyloid pathology, neurofibrillary tangles and synaptic and neuronal loss [3]. Amyloid-β peptide (Aβ) can deposit as extracellular senile plaques (SP) or cerebral amyloid angiopathy (CAA) surrounding brain vessels. On the other hand, tau dysfunction includes hyper-phosphorylation and aggregation of tau-protein in paired helical filaments, the major constituent of neurofibrillary tangles, which can cause neuron loss in AD [4,5]. Neuronal loss is a later step in AD evolution and it is the pathological feature that best correlates with illness duration and severity [6,7]. Whereas the vast majority of the AD cases are sporadic and the causes unknown [8], neuropathological features and clinical phenotype are largely the same as in familiar AD cases.

Apart from classical neuropathological features of AD, other alterations have been described, and the concept of neurodegeneration has expanded to accommodate early alterations that include oxidative stress or inflammation [9]. Following this idea, neuroinflammation also plays a significant role in neurodegeneration [10]. In this sense, AD brains suffer neuroinflammatory changes including alterations in morphology and distribution of microglia and astrocytes, or increased expression of inflammatory mediators [11,12,13]. While microglia’s response to injury is typically beneficial, it can go awry in cases of chronic injury and long-term inflammation, such as in AD. Thereby, microglia is chronically activated in the proximity of amyloid plaques and limits amyloid plaque growth [14] but, as the disease progresses, microglia fails to clear Aβ from brain parenchyma and ultimately contributes to the neurodegenerative process [13,15,16].

On the other hand, growing evidence has shown that alterations in the gut microbiota composition can alter normal brain function through the so-called gut–brain axis. Following this idea, the bidirectional communication between the central nervous system (CNS) and the gastrointestinal tract plays a key role in the pathogenesis of a variety of neuropsychiatric disorders and AD [17]. Moreover, regulation of gut microbiota has been implicated in the control of neuroinflammation and AD progression [18]. Additionally, in opposite direction, AD pathology could affect gut microbiota composition [17,19].

## 2. Prediabetes and Type 2 Diabetes Mellitus

Metabolic disorders include several pathologies. Among these, diabetes mellitus (DM) is one of the most prevalent diseases in the world. Moreover, type 2 diabetes (T2DM) is one of the leading causes of premature morbidity and mortality and its incidence increases with age [20,21]. The total number of people affected by DM is projected to rise up to 700 million in 2045 and about 90% of all the cases are of T2DM [22]. T2DM is characterized by an initial stage of insulin resistance or prediabetes, caused by the inability of the cells to fully respond to insulin, associated with blood glucose levels above the normal range but below the diabetes diagnostic threshold [23]. Currently, the most accepted position is that β-cell dysfunction, as impaired insulin secretion, is an independent abnormality that precedes dysglycemia, on a background of insulin resistance [24]. Although the development of this pathology shows high hereditability, T2DM seems to be caused by a combination of lifestyle and genetic factors [25]. In this sense, T2DM prevalence is increasing in children and younger adults, due to rising levels of obesity, sedentary lifestyle and inappropriate diet [26,27].

Systemic inflammation also plays an important role in insulin resistance and the pathogenesis of T2DM. The disease is associated to a pro-inflammatory state with a moderate excess in the production of cytokines such as IL-6, IL-1 or tumor necrosis factor (TNF), which hinder the interaction of insulin with its receptor and contribute to insulin resistance and diabetes [28]. On the other hand, gut microbiota also seems to play a role in metabolic disorders including obesity, prediabetes and T2DM by influencing glucose homeostasis and insulin resistance, as well as by affecting digestion of sugars and production of gut hormones that control this process [29,30,31,32]. Nevertheless, the strict control of glucose levels in the gut is not only mediated by hormonal control but also by gut-brain axis control [33]. Moreover, gut dysbiosis may affect intestinal permeability, as well as the metabolism of carbohydrates and their products [34,35]. Following this idea, T2DM patients show gut microbial dysbiosis, including reduced abundance of some butyrate-producing bacteria or increased opportunistic pathogens [36]. The decrease of short-chain fatty acids (SCFAs) may ultimately contribute to insulin resistance [37], that can be reverted by SCFAs administration [38]. In this sense, the restoration of gut microbiota can improve metabolic alterations, which position pre- and probiotics as an interesting option to prevent and/or treat prediabetes and T2DM [31,39].

## 3. AD-T2DM Relationship

Previous epidemiological studies have shown that T2DM is strongly associated with cognitive impairment and late-onset AD [40,41,42,43,44]. Both pathologies, AD and T2DM, share several pathophysiological features [45,46]. Whereas implicated mechanisms have not been fully elucidated [47,48] inflammation, diet or gut microbiota have been analyzed due to their possible role in the development of AD, as presented below.

### 3.1. Inflammation

Microglia responds to pathogens and modulates the immune response in the CNS [49]. Although brain neuroinflammation is considered a relevant player in neurodegenerative diseases and AD [50,51] a cross-talk between peripheral and central inflammation has also been described [52]. The dual role of microglia in AD has been largely addressed [53,54] and whereas microglia may limit SP growth [14], uncontrolled chronic inflammation and release of neurotoxic factors, including inflammatory mediators and reactive oxygen species, result in the exacerbation of AD pathology [55,56,57]. While brain inflammation is a hallmark associated with neurodegeneration in AD, peripheral blood inflammatory markers, such as IL-6, TNF-α or TGF-β are detected in AD patients as well [58]. Additionally, systemic inflammation plays an important role in the pathogenesis of prediabetes and T2DM [59], given that the adipose tissue can overproduce pro-inflammatory cytokines like TNF-α, which impairs insulin signalling, increasing the risk of insulin resistance [60]. Besides, TNF-α contributes to impair insulin signalling by increasing insulin receptor substrate-1 phosphorylation [61]. Altogether, it seems that T2DM, or even prediabetes, can cooperatively modulate the expression of brain pro-inflammatory cytokines in AD [12], and both the prediabetic state and T2DM, promote microglia activation in AD mice [62,63,64], supporting the role of the inflammatory process as a link between AD and T2DM.

### 3.2. Diet

Nutrition is considered a lifestyle factor that can modify the risk of dementia [65]. Nutrients reach the brain via the blood–brain barrier (BBB) by different mechanisms, such as facilitated diffusion or active transport [66]. Several studies have shown the role of specific nutrients, including folates and B12 vitamin, in brain volume loss in adult patients [67,68]. One of the most important effects of the diet is the ability to promote or control inflammation and therefore, neuroinflammation can be modified by a dietary pattern [69]. In this sense, Mediterranean diet (MD), considered an anti-inflammatory diet, is associated with preserved cognitive functions in elderly adults [70]. Ketogenic diet may improve neuropathological and biochemical changes associated to AD [71] as well as preserve cognition in patients with mild AD [72,73]. Likewise, studies in elderly patients have shown the neuroprotective effects of caloric restriction on memory [74].

On the other hand, T2DM onset can be improved by modifying lifestyle, including diet intervention [75]. Accordingly, healthy lifestyle with regular physical activity [76], high dietary fiber intake [77] and anti-inflammatory diets, like the MD, have positive effects on insulin secretion patterns [78]. Moreover, vegetarian diet also improves glycemic control in patients [79,80]. Hence, given the close relation between AD and T2DM, diet may also be an important link factor to modulate T2DM onset and AD risk.

### 3.3. Gut Microbiota

Gut microbiota encodes over three million genes producing thousands of metabolites with diverse roles, including extraction, synthesis, and absorption of many nutrients and metabolites [81]. Gut microbiota also shows an immune function against pathogenic bacteria colonization and promotes intestinal epithelium integrity [82,83,84]. It implies a wide range of microorganisms, being the dominant phyla *Firmicutes* and *Bacteroidetes*, which represents 90 percent of gut microbiota. *Actinobacteria*, *Proteobacteria*, *Fusobacteria*, and *Verrucomicrobia*, are also representative groups [85]. The human gut microbiota suffers taxonomic and functional modifications in each part of the gastro-intestinal tract. This leads to several variations in the same individual, due to age and environmental factors, although microbiota functions are preserved among individuals [86]. Additionally, modifications in the diversity or structure of gut microbiota known as dysbiosis, can affect metabolic activities, resulting in metabolic disorders, like obesity and diabetes. Consequently, dysbiosis may play a crucial role in the development of T2DM [32,87]. In this sense, it has been described that type 1 diabetes and T2DM patients show microbiota dysbiosis [88], which contributes to the onset and maintenance of insulin resistance [37]. Additionally, dietary habits can influence gut microbiota composition. In this sense, the Western diet (WD), a high-fat/high-sugar diet and low fiber intake, induces dysbiosis and it is associated with obesity and metabolic diseases [89]. On the other hand, studies using the MD have shown the benefits of modifying the intestinal microbiota in obese subjects for the prevention of the development of T2DM [89].

On the other hand, the brain-gut-microbiota axis also relates microbiota with brain pathologies like AD and Parkinson’s disease [90,91]. Thus, AD patients present decreased microbial diversity, including reduced *Firmicutes*, and increased *Bacteriodetes* [92]. Furthermore, dementia patients with amyloidosis have increased pro-inflammatory cytokines levels in blood accompanied by increased pro-inflammatory intestinal bacteria [93]. It should be noted that several genera of gut microbiota can produce or metabolize neurotransmitters, such as γ-aminobutyric acid (GABA) [94], which play a relevant role in brain aging [95]. These findings position gut bacterial communities as a target for therapeutic intervention. Moreover, given the close relation between AD and metabolic alterations, gut microbiota could provide a relevant link between both pathologies. In this sense, undigested foods, microbes, endotoxins and immune response cause chronic inflammation that may ultimately contribute to alter the BBB integrity and trigger neuroinflammation [96]. Interestingly, a recent study shows that obesity, highly present in T2DM patients, impairs short-term and working memory through gut microbial metabolism of aromatic amino acids [3], offering a new venue that relates metabolic alterations and cognitive impairment.

## 4. Diet, Inflammation and Gut Microbiota in AD Models

Mouse models offer a good approximation to study human gut microbiota and their implications in diseases and inflammatory processes [97,98]. Despite appreciable differences between the intestinal tract of mice and humans [97], they share anatomical and physiological similarities [98]. Interestingly, whereas there are many variations at lower taxonomic level, gut microbiota composition shows similarities at a higher taxonomic level [99]. According to KEGG (Kyoto Encyclopedia of Genes and Genomes) database [100], when microbiome is analyzed dissimilarities are limited at functional level and mouse and human share 95.2% of the KEGG orthologous groups. Nevertheless, mice and humans only share 4.0% of the genes [99]. The mouse microbiome seems to have specifically enriched pathways involved in membrane transport, biodegradation and amino acids metabolism [99,101].

Diet and housing conditions may markedly influence the composition of the gut microbiome [84,99,102] together with the age [103], gender [104] and the genotype of the mice [105,106]. However, gut microbiome in mice is mainly composed by bacteria belonging to the phylum *Bacteroidetes*, followed by phyla *Firmicutes, Deferribacteres* [99,101] and *Proteobacteria* [107], although the actual order depends on the studies [99].

As previously stated (Section 3.3. Gut microbiota), AD patients [108,109] and AD preclinical models [110,111] present significant dysbiosis and alterations in the composition of the microbiome. Concretely, APP/PS1 mice dysbiosis leads to alterations in metabolic and cellular pathways, including the phosphatidylinositol signaling system, limonene and pinene degradation, as well as butanoate/pyruvate metabolism [111]. Microbiome and their secretory products, such as lipopolysaccharide (LPS), are toxins that play a key role in the immune response [112,113] and are involved in microglial activation [114]. SCFAs (acetate, propionate and butyrate) are products of anaerobic bacteria fermentation of dietary fibers in the intestine. SCFAs are relevant gut-microbiota mediators of inflammation and immune response [115,116], they have a protective effect on epithelial cells [117] and decrease Aβ fibrillation *in vitro* [118]. Propionate, a potent neurotoxic agent, is increased in 3xTg-AD mice by 6 months of age [119]. On the other hand, the concentration of SCFAs, including butyric and isobutyric acids, tend to be lower in both feces and brains from 3xTg-AD mice [111,119]. Likewise, gut microbiome can modulate the inflammatory response in both, the intestinal tract and the brain, through gut-brain axis [115,120,121]. Besides, gut microbiota is critical for microglia maturation [122,123] and activation in the CNS [122,124]. Activated microglia cells are significantly increased in the brains from 11-month 3xTg-AD mice [119] and, in germ-free APP/PS1 mice, a reduction of cortical microglia cells has been reported at 3.5 and 8 months of age [124]. Additionally, pro-inflammatory cytokines IL-6 or IL-1β, as well as lipocalin-2 or TNF-α expression levels are increased in the colon of APP/PS1 mice by 3 and 6 months of age [125]. Similar results have been observed in SAMP8 mice, a spontaneous AD model, with colonic inflammation and high IL-1β levels [126]. Moreover, IL-1β was increased, while IFN-γ, IL-2 and IL-5 levels decreased in the brain from aged APP/PS1 mice [124]. In addition, a similar cytokines profile can be observed in plasma from 14-month 3xTg-AD mice, with higher levels of pro-inflammatory cytokines such as IL-12p40, whereas IL-1α, IL-1β, IL-3, IL-5, IL-6, IL-12 p70, IL-17, TNF-α, IFN-γ, CCL2, CCL3, CCL5, CCL11 and GM-CSF are decreased [127].

Gut-brain axis and gut microbiome seem to be fundamental for the load of Aβ in the brain [124,128]. Previous studies have shown that germ-free APP/PS1 (amyloid precursor protein/presenilin 1) mice, have lower levels of Aβ, mainly Aβ42, in brain and plasma, whereas colonization by microbiota from aged APP/PS1 mice results in increased levels of Aβ, supporting a close cross-talk between gut and brain in AD [124]. Moreover, APP/PS1 mice on long-term antibiotics treatment (gentamicin, vancomycin, metronidazole, neomycin, ampicillin, kanamycin, colistin and cefaperazone) show lower Aβ plaque deposition and higher soluble Aβ production, favoring Aβ42 formation in the cortex and hippocampus [129]. In addition, wildtype fecal solution administered to APP/PS1 mice, previously treated with antibiotics, also results in lower Aβ deposition and better cognitive performance [130]. Similar outcomes have been described in ADLP^APT^ mice after transferring fecal microbiota from wildtype mice for 4 months. Treated ADLP^APT^ mice show lower SP burden and lower Aβ levels [128].

Differences in gut microbiota are observed when wildtype and AD mice are compared (Table 1) [107,111,131]. Previous studies have reported lower bacterial diversity and more similar bacteria composition when young AD mice are compared with wildtype counterparts [90,107,132], while further differences are shown as the age of the animals increase [90]. Following this idea, gut microbiota from young APP/PS1 mice [107] and 5xFAD mice [131,133] are similar to that observed in wildtype animals. No significant differences in either α- or β-diversity have been observed in 5xFAD mice at 9–10 weeks old [133], although α-diversity is lower in these mice by 6 and 12 months of age [134]. Nevertheless, α-diversity, that conveys the diversity of species within a single sample, is significantly higher in wildtype mice compared to APP/PS1 mice by 9 months of age [135], although other studies have reported higher α-diversity in the microbiota of APP/PS1 mice at the same age [90]. It also seems that α-diversity is diminished with aging in APP/PS1 mice, as an indication of a worse health status of the host with aging [132]. On the other hand, β-diversity shows significant differences in the microbiome composition of APP/PS1 mice by 2, 6 [90] and 9 months of age [135] when compared with their wildtype counterparts. So, as disease progresses a marked temporal shift in the taxonomic distribution of principal phyla is detected, and the *Firmicutes*/*Bacteroidetes* ratio increases in APP/PS1 [107] and 5xFAD mice [131,134] in an age-dependent manner. An increase in *Firmicutes*/*Bacteroidetes* ratio is also observed when bacterial extracellular vesicles are analyzed in the serum of 8-month APP/PS1 mice [136]. Nevertheless, the ratio *Firmicutes*/*Bacteroidetes* is decreased in APP^NL-G-F^ mice at this age [137], underlying the complexity of these studies.

Gram-negative phylum *Bacteroidetes*, that are the main producers of acetate and propionate [141], seem to steadily increase with aging in both APP/PS1 and wildtype animals, but they are less abundant in APP/PS1 mice, being families *Rikenellaceae* [90,107] and *Bacteroidaceae* (genus *Prevotella*) [132] significantly lower in APP/PS1 mice [90]. A similar pattern is observed in 5xFAD mice at 6 and 12 months of age [134]. Oppositely, genus *Prevotella* seems to be increased in 3xTg-AD mice by 11 months of age [119].

On the other hand, gram-positive phylum *Firmicutes*, the main butyrate producing-bacteria [141], increases with age and has higher presence in APP/PS1 animals than in wildtype mice by 24 months of age [107]. Similar outcomes have been reported in 5xFAD mice by 9 weeks of age [139] and in 15-month tg2576 mice [138]. This increase in *Firmicutes*, observed in AD models, is mainly due to members of the family *Erysipelotrichaceae* [107] and detected dysbiosis develops an immunogenic reaction [142] that is correlated with increased levels of proinflammatory cytokines, such as TNFα [143]. By contrast, a decrease in the number of SCFAs and butyrate-producing bacteria is found in AD models. So, families *Ruminococcaceae* and *Clostridiaceae* (class *Clostridia*) are significantly decreased in 8–12-month APP/PS1 mice, accompanied by altered butanoate/pyruvate metabolism [111]. There is a positive correlation between the abundance in family *Ruminococcaceae* and the concentration of hypoxantine [144], an intermediate metabolite of purines which has a critical role in the maintenance of the intestinal barrier function [145] and it is altered in 6-month tg2576 mice [138]. Moreover, genus *Ruminococcus* (family *Ruminococcaceae*) is significantly lower in 8-months APP/PS1 mice, when spatial learning is aggravated in these mice [132]. Conversely, family *Ruminococcaceae* is increased in 5xFAD mice by 6 and 12 months of age [134] and it has been reported that *Clostridium leptum* is transiently increased in feces of 5xFAD mice at 9 weeks old, as a reflection of a compensatory mechanism in the early phases of AD [139].

In addition, 5xFAD mice have increased *δ-*, *γ-* and *ε-Proteobacteria* by 6 months of age, while at 12 months *β-* and *δ-Proteobacteria* are increased and *ε-Proteobacteria* is decreased when compared with wildtype counterparts [134]. In line with these observations, genus *Desulfovibrio* is reported to be highly increased in 5 to 9 month-old APP/PS1 mice microbiome [90,111,130,132]. As part of this phylum, the genus *Sutterella* (class *β-Proteobacteria*) is significantly increased in APP/PS1 mice in an age-dependent manner [107] and similar outcomes have been reported in 5xFAD mice at 12 months old [134]. *Sutterella spp.* specifically induces mild or negligible inflammatory responses and does not alter epithelial monolayer integrity [146], although they are increased in feces from patients with cognitive disorders [147]. Nevertheless, their immunomodulatory role is not fully understood [148]. *Enterobacteriaceae* is also increased in the microbiome of APP/PS1 mice by 6 months of age [90]. On the other hand, phylum *Verrucomicrobia*, and its only known genus, mucin-degrading bacteria *Akkermansia*, down-regulates the expression of the pro-inflammatory cytokines TNF-α and IFN-γ in the colon [149]. *Akkermansia* is significantly increased in APP/PS1 mice along lifespan, starting at 2 months of age [90] up to 7 [130] and 12 months of age, when it is dramatically increased [111]. In 5xFAD mice, the presence of this phylum is limited in young mice, when compared to their wildtype counterparts [139], while it increases by 12 months of age [134].

The overall dysbiosis detected in AD mice (Table 1) leads to a proinflammatory microbiome that contributes to the inflammatory process [150]. As previously stated, dietary habits strongly influence the gut microbiota composition [84,102]. Therefore, new strategies try to favor a healthy microbiome through the administration of pre- and probiotics supplements as a possible treatment for AD [151]. In line with this, 3xTg-AD mice orally fed with probiotic SLAB51 (*Streptococcus*, *Bifidobacteria*, *Lactobacilli*) presented higher levels of *Bifidobacterium spp*. and reduced levels of *Campylobacterales*, along with an increase of anti-inflammatory SCFAs and cytokines that down-regulate inflammatory response. A reduction of amyloid load is also detected, accompanied by an improvement of cognitive alterations [152]. In other study, FRAMELIM^®^ (*Bifidobacterium*, *Lactobacillus*, vitamins A, D and omega 3 fatty acid) administration to APP/PS1 mice successfully reduced brain Aβ burden, despite lowering the abundance of butyrate-producing bacteria [153]. Antioxidants, such as PW5 have also provoked a shift in gut microbiota composition from APP/PS1 mice, making them more similar to wildtype counterparts, while reducing SP in the hippocampus [154]. Although it has been described that pre- and probiotics have little effect on the microbiome of healthy population [155], the complexity of this research venue may require more in depth studies to fully elucidate their specific roles on microbiome and inflammation [156].

Caloric restriction diets have also been associated with an increase of dendritic spines in the dentate gyrus and cognitive protection in mice [157]. Intermittent fasting (IF) has also shown beneficial effects on neuropathological characteristics and cognitive deficits in AD [158,159,160,161]. IF leads to ketogenic metabolism, increasing β-hydroxybutyrate levels in serum and CSF [162]. Interestingly, β-hydroxybutyrate protects against Aβ toxicity [163,164] and protects hippocampal murine neurons [165,166]. Whereas previous studies have reported no significant effects of IF on neuroinflammation, synaptic plasticity or cognitive function in 6-month 5xFAD mice [167], it has also been shown that IF restores aquaporin-4 polarity, reduces SP deposition and ameliorates cognitive dysfunction in APP/PS1 mice [168]. Cognitive impairment is also improved in APP^NL-G-F^ mice after IF for 9 months, whereas no significant effect is observed in amyloid load [169]. It has also been reported that IF for 3 months may increase brain-derived neurotrophic factor (BDNF) and neuronal differentiation in the dentate gyrus from 3xTg-AD mice [170]. In line with these observations, it has been shown that ketogenic diet reduces amyloid deposition and tau pathology, improving learning and memory tasks in a 3xTg-AD mouse model [171].

## 5. Diet, Inflammation and Gut Microbiota in T2DM Models

As previously stated, T2DM includes a heterogeneous group of metabolic disorders, characterized by hyperglycemia, and influenced by both genetic and environmental factors [172,173], in which lifestyle and dietary habits are strongly implicated [174]. The most commonly used T2DM mouse models are based on leptin signaling impairment [175], including db/db and ob/ob mice. Diet-induced models are also widely used, and different types of high-fat diet (HFD) are regularly administered to induce prediabetes, as well as to resemble the effects of WD [176,177,178,179,180,181]. HFD feeding increases body weight, leptin and insulin levels as well as insulin resistance in rodents [176,182,183]. Interestingly, the effects of HFD administration seems to be influenced by gut microbiota and the genetic background of the animals under study [184].

Inflammatory cells including neutrophils, abnormal lymphocytes and monocytes are increased in db/db mice [185]. In line with these observations, CD68 mRNA, as a macrophage marker, is highly expressed in white adipose tissue in these mice [186]. In the CNS of db/db mice, microglia cells are also increased [63] and exhibit greater reactivity to LPS stimulation [187]. In fact, LPS serum levels are markedly increased in db/db mice [188]. Cytokines profile is also affected and db/db mice and previous studies have shown an increase of pro-inflammatory cytokines, including IL-1β and TNF-α in serum [185], hippocampus [187] and liver [189]. CCL2, a chemokine that recruits monocytes and macrophages, is highly expressed in epididymal adipose tissue of db/db mice [186], whereas the expression of nucleotide-binding-oligomerization domain type-1 (NOD1) is suppressed [186]. Inflammation also affects the vasculature and inflammatory markers such as monocyte chemotactic protein-1 (MCP-1), vascular cell adhesion molecule-1, (VCAM1), intercellular adhesion molecule-1 (ICAM1) or E-selectin are increased in aortic and carotid vessels from db/db mice [190]. Likewise, oxidative stress markers, as like 3NT-positive protein, are increased in serum [191].

The ob/ob mouse also presents chronic inflammation of the adipose tissues which contributes to the metabolic pathogenesis of obesity and insulin resistance [177,192,193]. Cytokines and chemokines like TNF-α, IL-1β, IL-6, and CCL2 are overexpressed in visceral adipose tissue [177,194]. In line with these observations, elevated TNF-α levels lead to a decrease in Azgp1 mRNA and protein levels in adipose tissues of ob/ob mice [177,192]. Additionally, IL-6, TNF-α [195,196,197], IL-1β, IL-10 [193] and plasminogen activator inhibitor-1 (PAI-1) are increased in the adipose tissue and the spleen of ob/ob mice [195,197]. IFN-γ expression decreases in ob/ob mice [193] and other studies have reported lower adiponectin values in the adipose tissue and in the spleen [193,195]. Moreover, LPS levels are higher in plasma from ob/ob mice [198] and in C57BL/6NTac mice on HFD [174]. Additionally, NF-kB levels are elevated in the brain of ob/ob mice [196]. Other markers, such as CD11 and CD86 are increased in the brain and visceral fat tissue, whereas the expression of CD206 decreases [196,197]. In line with these observations, oxidative stress is also severely affected in ob/ob animals and increased protein oxidation and lipoperoxidation are observed, accompanied by lower activity of antioxidant enzymes in kidney and heart [195].

As previously pointed out, diet is also an experimental approach to reproduce metabolic complications and mice fed a WD show infiltration of lymphocytes and neutrophils in the liver, indicating the presence of steatohepatitis, and increased TNF-α-positive macrophages [199]. Animals on WD overexpress pro-inflammatory markers such as IL-1β, IL-6, TNF-α, FOXP3, SAA1, CCL17, CCL20 and ICAM-1 in the brain [185,199] and CD68 expression is also increased in these mice [186]. The expression of NOD-1 is suppressed in mice fed with WD [186]. Following these observations, animals on HFD show an increase of crown-like structures and up-regulation of pro-inflammatory genes, indicating adipose tissue inflammation associated with diet [200]. Interestingly, whereas adipose tissue inflammation is observed after administration of HFD for 24 weeks, hepatic inflammation is not detected up to 40 weeks on HFD [200]. Moreover, adipose inflammation seems to contribute to insulin resistance more severely than hepatic inflammation [200]. In addition, He et al. found out that the phenotypic switch in adipose tissue from anti-inflammatory to pro-inflammatory macrophages is detected after 5 weeks on HFD, while metabolic inflammation is noted after 9 weeks [201]. An overall increase of pro-inflammatory cytokines such as TNF-α, IL-6, leptin and TGFβ1 is detected in the liver of HFD-fed mice for 17 weeks [202] and up to 40 weeks [200]. Moreover, other markers, such as MCP-1, PAI-1 and CCL2 are increased in adipose tissue of mice on HFD for 9 weeks [176] or up to 17 weeks [202].

The gut microbiota in T2DM mice seems to be highly dependent on the genetic background and age of the animals under study [203,204,205] (Table 2). In ob/ob and db/db mice *Bacteroidetes* and *Firmicutes* are the dominant phyla in the gut microbiota [203,206], followed by *Proteobacteria*, *Tenericutes*, *Actinobacteria* and *Cyanobacteria* [203,207]. Other less abundant phyla found in these mice are *Acidobacteria*, *Deferribacteres*, *Gemmatimonadetes*, *Lentishphaerae* and *Verrucomicrobia* [205,208]. Additionally, the ratio *Bacteroidetes*/*Firmicutes* is higher in db/db mice, than on C57Bl/6J mice [205]. On the other hand, family *Muribaculaceae* is reduced in db/db mice by 8 months of age [209], while other studies have reported that genus *Rikenella* (family *Rikenellaceae*) is increased in db/db mice by 6 months of age [210]. It also seems that genera *Paraprevotella* and *Alloprevotella*, (family *Prevotellaceae*) are increased in db/db mice by 6 months of age, while the genus *Alloprevotella* decreases by 8 months of age [209,211]. Families *Enterococcaceae* and *Planococcaceae* (class *Bacilli*) [211], as well as genus *Klebsiella* (*Enterobacteriaceae*) are also increased in db/db mice by 6 months of age [211].

Interestingly, previous studies have suggested that T2DM pathology can be ameliorated by administering pre- and probiotics that may induce modifications in the microbiome [213,214,215]. Following this idea, treatment with *Lactobacillus acidophilu*s reduces pro-inflammatory cytokines, including IL-8, TNFα, and IL-1β in liver and colon of T2DM mice [87]. Prebiotic inulin modulates gut dysbiosis, increasing phylum *Cyanobacteria* and genus *Bacteroides*, that positively correlate with higher levels of IL-10 at early stages of T2DM, and negatively correlate with IL-6 and TNF-α in db/db mice [216]. A reduction of *Ruminiclostridium_6*, *Deferribacteres* and *Tenericutes* is also detected in inulin-treated T2DM mice, that correlates with IL-6, TNF-α and IL-17A levels [216]. Additionally, diet supplements such as polysaccharides from mulberry fruit [217] and *Alpinia oxyphyla* Miq. extract [218], show beneficial effects at microbiome level in db/db mice. Likewise, supplementation with *Alpinia oxyphyla* Miq. extract increases *Akkermasia* spp. while *Helicobacter* spp. is significantly reduced in db/db mice [218]. Zhang et al., have reported that berberine and metformin treatment reduces LPS serum levels, relieve intestinal inflammation and repair intestinal barrier structures [188]. This treatment modifies gut microbiome in db/db mice, increasing the number of SCFAs producing bacteria and *Lactobacillus* and *Akkermansia,* while decreasing opportunistic pathogens [188]. On the other hand, sodium-butyrate supplementation also ameliorates diabetic inflammation in db/db mice, decreasing LPS and HbA1c. It also decreases the ratio *Firmicutes*/*Bacteroidetes* [185]. In mice on HFD, 15 weeks treatment with baicalin, a flavonoid used in traditional Chinese medicine, rebalances the microbiota and SCFAs-producing agents specifically, to protect the intestinal system. Baicalin seems to regulate metabolic and immunological processes implicated in T2DM [219]. In line with these observations, another study, using the JinQi Jiangtang (*Coptis chinensis*, *Astragalus membranaceus* and *Lonicera japonica*), has reported reduced levels of TNF-α and IL-6, ameliorating the inflammatory response and regulating the intestinal microbiota [205]. Diabetic mice treated with *A. membranaceus* also show increased microbial diversity and enrichment of the bacterial *Firmicutes spp.*, *Acidobacteria* and *Gemmatimonodetes* [212].

On the other hand, db/db mice on IF show a reduction of NFκB activation and decreased LPS levels, associated with a significant improvement of cognitive function, limited neuroinflammatory response, restructured gut microbiota and metabolites [27,220] and reduced insulin resistance [207]. Other studies in db/db mice on IF regimen for 28 days have reported a reduction of *Enterococcus*, *Streptococcus*, *Enterococcaceae*, *Arthromitus*, *Rummeliibacillus* that cause inflammation, while increasing *Lactobacillus*, accompanied by the attenuation of behavioral alterations [207].

## 6. Microbiota, Inflammation and Diet in Preclinical Mixed Models in AD-T2DM

The close relation between AD and T2DM has been largely documented [9,221,222,223,224]. While still limited, previous studies have reported the presence of AD-like pathological features related to alterations in the microbiota [207,212,216,225,226] and the inflammatory response [227] in animal models that reproduce different metabolic alterations, suggesting that the gut-brain axis may explain the molecular similarities that link AD and T2DM [228,229]. Following this idea, it has been described that chronic bacterial infections are associated with amyloidosis, one of the pathological features shared by AD and T2DM [227].

Apolipoprotein E (APOE) 4 gene allele, the highest genetic risk factor for developing late-onset AD, is associated with increased Aβ load, neuroinflammation and alterations in brain insulin signaling [230,231,232,233]. Studies on E4FAD mice, obtained for breeding 5xFAD mice with homozygous APOE4 mice, have shown that soluble Aβ42 levels and total Aβ levels are higher when compared to E2FAD and E3FAD mice. Moreover, SP deposition is higher and more compact in E4FAD mice, leading to neuroinflammation in the hippocampus [234]. ApoE^-/-^ mice on HFD have also shown higher APP expression [235] and increased Aβ levels [236]. No significant differences have been observed in the expression of doublecortin or PSD95 in ApoE^-/-^ and APOE4 mice on HFD, indicating that HFD does not affect neurogenesis and synaptic plasticity [237]. Some studies have shown that WD administration to adult APP/PS1 mice does not result in increased SP deposition or soluble Aβ levels compared to animals on ND [238]. Nevertheless, the vast majority of available bibliography supports the deleterious effect of HDF administration. HFD increases hippocampal Aβ deposition in 5xFAD mice with no significant effect on memory [239]. APP/PS1 mice on HFD present a lower percentage of spines and deterioration of newborn neurons when compared with wildtype mice on normal diet (ND) [240]. Similarly, an exacerbation of synaptic loss has been detected in APP/PS1xdb/db mice [241]. Other studies have reported increased amyloid and tau pathologies, vascular damage and cognitive impairment in APP/PS1 mice on HFD [64]. Even short-term HFD [242] increases tau phosphorylation [236] and cognitive impairment is also increased 3xTg-AD on HFD [243]. Cognitive deficits might be due to loss of synaptic plasticity, since a reduction of matrix metalloproteinases 2/9 activity accompanied by a decrease in the expression of the BDNF gene have been reported [238].

An overlap in the inflammatory mechanisms has also been described in AD and T2DM [244]. Previous findings confirmed that the microglial receptor for advanced glycation end products in T2DM may mediate microglial activation and neuroinflammation AD brains [244,245]. Increased neuroinflammation might not be exclusively related to HFD, since wildtype mice on HFD do not show a significant increase in the number of activated glial cells in the hippocampus, supporting a synergistic contribution when AD and metabolic disease are set together [240], as regularly observed in the clinic. MIP-1α, IL-1β, and IL-17 are altered in the cortex from APP/PS1 mice on HFD that also show increased IL-1β and TNF-α, accompanied by gliosis and disrupted neurogenesis in the hippocampus [12,240]. Higher levels of circulating blood monocytes, MCP-1, and ox-LDL are also observed in plasma from these mice, indicating severe systemic inflammation in these animals [238]. Interestingly, IL1-β, IL-12p70 and IL-13 correlate with insulin levels in APP/PS1xdb/db mice and IL-3, IL-17, IL-4, and KC (CXCL1) are also affected in these mice [12], suggesting that specific cytokine profiles are associated with AD and T2DM. In line with these observations, microglial activation is also increased 3xTg-AD on HFD [246].

An exacerbated production of pro-inflammatory cytokines, such as IL-1, IL-6 or TNF-α is observed in APOE4 mice [247]. Moreover, FAD mutations induce neuroinflammation in the hippocampus of APOE4 mice, by inducing the expression of genes for chemokine ligand 4, low-affinity Fc IgG receptor 4, chemokine 10 of the CXC motif, and α-integrin increased [248]. On the other hand, ApoE^-/-^ and APOE4 mice show significantly more inflammation in CA1 when fed with ND than wildtype mice, but these mice treated with HFD show decreased CD68 immunoreactivity in the hippocampus [237]. Furthermore, microbiome related inflammation may play a role at this level [249,250,251] since wildtype and ApoE^-/-^ mice on HFD present higher LPS plasma levels and a higher expression of cytokines in the colon and hippocampus [235]. LPS induced endotoxemia in APOE4 mice results in an increase of plasma TNFα and G-CSF when compared with APOE3 mice. Besides, CD8^+^ T cell apoptosis is observed in APOE4 mice after LPS injection [249].

Diet-induced intestinal dysbiosis produces an imbalance in the microbiota that can have inflammatory consequences in the AD brain [106]. Interestingly, alteration of brain function and gut microbiota have been related to HFD administration during gestation [252]. Therefore, the disbalance in the composition of the gut microbiota could explain the altered eating behavior, memory impairment and abnormal exploratory performance of the offspring from mothers on HFD. Moreover, these studies may support the implications of early-life alterations in later development of neurodegenerative complications [253].

Specific assessment of 3xTg-AD mice on HFD showed a severe lack of the families *Muribaculaceae*, *Peptococcaceae* (genus *rc4.4*), *Dehalobacteriaceae*, accompanied by an excess of genus *Clostridium* (Table 3) [106]. This study confirms that HFD and genetic predisposition to neurodegenerative diseases share abnormalities in the microbiome and metabolome that are unfavorable for brain health. On the other hand, microbiota alterations associated with APOE have been found at 4 and 6-months of age, including changes in *Prevotellaceae*, *Rikenellaceae, Gastranaerophilales*, *Lactobacillaceae*, *Peptococcaceae*, *Erysipelotrichaceae* (genus *Turicibacter*), *Desulfovibrionaceae* and *Mycoplasmataceae* (genus *Mollicutes*) [250]. Moreover, E4FAD mice had a greater α-diversity but reduced relative abundance of beneficial gut microbiota (*Prevotella* and *Lactobacillus*), accompanied by an increase of pro-inflammatory microbiota (*Escherichia*, *Turicibacter* and *Akkermansia*) (Table 3) [248]. However, *Lactococcus*, that has been implicated in a better performance in behavioral tests, is not present in ApoE^−/−^ animals [235].

Caloric restriction has been considered an experimental diet that can lengthen the lifespan in various animal models [254,255,256] and may help to establish a balanced architecture of the intestinal microbiota with beneficial effects on the health of the host by reducing the intestinal antigen load [256]. Other diet strategies to ameliorate AD symptoms are based in prebiotics such as inulin that stimulates the growth and activity of SCFAs-producing bacteria. Inulin administration also reduces proinflammatory gene expression in the hippocampus and Aβ deposits in E4FAD mice [248]. It also contributed to a larger size of the caecum, improving the microbial content, even before the development of the Aβ pathology in E4FAD mice. An improvement in systemic metabolism associated to the modulation of the intestinal microbiome has also observed in this model [248]. Although further studies would be necessary to understand the functional and morphological characteristics mediated by inflammation, microbiota, and diet in mixed preclinical models of AD-T2DM, observations based on experimental animal models may contribute to the development of optimal techniques and therapies to reverse the complications associated with AD and metabolic alterations.

## 7. Common Microbiota Alterations in AD, T2DM and Mixed Preclinical Models

AD and T2DM share common pathological features that include impairment in insulin signaling, alteration of amyloid and tau pathologies or vascular damage that have been largely revised before (Mejido, Penny et al., 2020; Goncalves et al., 2019; Ferreira et al., 2019; Vieira et al., 2018). At inflammatory level, as stated in this review and in previous studies, proinflammatory cytokines are favored in AD, T2DM and mixed preclinical models. Among others, it has been shown that IL-1β is increased in the brain from aged APP/PS1 [124] as well as in APP/PS1xdb/db mice [12]. IL-1β and TNF-α are also increased in the brain from db/db mice [187] as well as in APP/PS1 mice on HFD [12,240].

Whereas the role of the gut microbiota as a feasible connection between both diseases remain largely unexplored, some common links have been reported (Graphic Abstract). *Bacteroidetes* and *Firmicutes* are the dominant phyla in AD, T2DM and mixed models [85,203,206]. *Bacteroidetes* (family *Muribaculaceae*) is reduced in db/db mice at 8 months of age [209] and 3xTg-AD mice on HFD also show a severe lack of *Bacteroidetes* [106]. This phylum (family *Prevotellaceae*) is also decreased in E4FAD and db/db mice at 8 months of age [209,211,248]. *Actinobacteria* (family *Coriobacteriaceae*) has been reported to be increased both in APP/PS1 [132] and in db/db mice [205], while the *Bacteroidaceae* family seems to be less abundant in different AD and T2DM preclinical models [90,132,134,204,209,210,211,257]. Nevertheless, other studies have reported increases in the *Bacteroidaceae* family, both in AD [119] and T2DM animals [204,209,210,211]. Phylum *Firmicutes* (family *Ruminococcaceae,* genus *Ruminococcus*) has been reported to be increased in AD [134] as well as in different T2DM preclinical models [203,205,206]. The *Clostridiaceae* family is transiently increased in the feces of 5xFAD mice at 9 weeks [139]. Similarly, it is increased in ob/ob mice at 8 weeks of age [257] and an excess of the genus *Clostridium* is observed in 3xTg-AD mice on HFD [106]. Other studies have reported that the *Lachnospiraceae* family is more abundant in AD preclinical models (3 month-old APP/ PS1 mice and 5xFAD mice at 6 months of age) [90,134] as well as in metabolic disease preclinical models (both after HFD administration and in 15-17 week old ob/ob mice) [203,206]. Likewise, the family *Lactobacillaceae* has been shown to be increased in AD mice (6 month-old Tg2576 and 11 month-old 3xTg-AD mice) [119,138] and similar outcomes have been reported in db/db mice at 14 weeks of age [206]. Other alterations include increases of the *Enterobacteriaceae* family in db/db and AD mice, including 6 month-old APP/PS1 [90] and E4FAD mice [248]. The *Desulfovibrionaceae* family (Genus *Desulfovibrio*) is also increased in 5–9 month-old APP/PS1 [111,130,132] and 6-12 month-old 5xFAD mice [134]. Similar outcomes have been reported in 12-week-old db/db mice [205] and after HFD administration [206]. It has also been shown that family *Helicobacteraceae* is increased in 3–8 month-old APP/PS1 mice, 6 month-old 5xFAD mice and after HFD administration [132,134,206], supporting similar changes in AD and metabolic disease models. Interestingly, previous studies suggest that AD and T2DM pathologies can improve through the treatment of pre-and probiotics [87,151,152,153,248,258], caloric restriction diets [27,157,207,220] or antioxidants [154], helping to regulate intestinal dysbiosis and reduce the inflammation.

## 8. Conclusions

The extensive review of available bibliography shows that gut microbiota composition, and the brain-gut axis are affected in AD, T2DM and mixed models of AD and metabolic disorders, affecting inflammation through products of bacterial metabolism, like SCAFs. Proinflammatory cytokines are widely altered in mouse models of both diseases. Microbiota is highly affected as diseases progress, and alterations in *Actinobacteria, Bacteroidetes* or *Firmicutes* phyla, among others, are commonly observed in animal models harboring AD, T2DM or AD and metabolic disease. All these observations based on preclinical models have contributed to further understand the key role of the gut-brain axis in both pathologies. Nevertheless, conflicting results have also been reported and therefore further studies would be necessary to fully understand the relation between diet, microbiota and inflammation in AD and T2DM.

## Figures and Tables

**Table 1:** Alterations of gut composition in AD preclinical models.

Phylum	Class	Family	AD Mice	Ref.
*Actinobacteria*	*Actinobacteria*	*Turicibacteriaceae*	= in APP/PS1 mice at 6–24 months of age	[107]
		*Coriobacteriaceae*	↑ in APP/PS1 mice at 6–8 months of age	[132]
*Bacteroidetes*	*Bacteroidia*	*Rikenellaceae*	↓ in APP/PS1 mice at 6–24 months of age	[90,107]
		*Bacteroidaceae* (Genus *Prevotella*)	↓ in APP/PS1 mice at 3–6–8 months of age	[90,132]
			↓ in 5xFAD mice at 6–12 months of age	[134]
			↑ in 3xTg-AD at 11 months of age	[119]
*Firmicutes*	*Erysipelotrichia*	*Erysipelotrichaceae*	↑ in APP/PS1 mice at 24 months of age	[107,111]
	*Clostridia*	*Ruminococcaceae*	↓ Genus *Ruminiclostridium* in tg2576 mice at 6–15 months of age	[138]
			↓ Genus *Ruminococcus* in APP/PS1 mice at 6–8 months of age	[111,132]
			↑ Genus *Ruminococcus* in 5xFAD mice at 6–12 months of age	[134]
		*Clostridiaceae*	↓ *Butyricicoccus pullicaecorum* in APP/PS1 mice at 8–12 months of age	[111]
			↑ transiently *Clostridium leptum* in 5xFAD mice at 9 weeks of age	[139]
		*Lachnospiraceae*	↑ in APP/PS1 mice at 3 months of age	[90]
			↑ in 5xFAD mice at 6 months of age	[134]
	*Bacilli*	*Lactobacillaceae* (Genus *Lactobacillus*)	↑ in Tg2576 at 6 months of age/= at 15 months of age	[138]
			↓ in APP/PS1 mice at 8 months of age	[140]
			↑ in 3xTg-AD mice at 11 months of age	[119]
			↓ in 5xFAD mice at 6 months of age	[134]
*Proteobacteria*	*β-proteobacteria*	*Sutterellaceae* (Genus *Sutterella*)	↑ in APP/PS1 mice at 6 months of age	[107]
			↑ in 5xFAD mice at 6–12 months of age	[134]
	*δ-proteobacteria*	*Desulfovibrionaceae* (Genus *Desulfovibrio*)	↑ in APP/PS1 mice at 5–9 months of age	[111,130,132]
			↑ in 5xFAD mice at 6–12 months of age	[134]
	*ε-proteobacteria*	*Helicobacteraceae*	↑ in APP/PS1 mice at 3–6–8 months of age	[132]
			↑ in 5xFAD mice at 6 months of age/↓ at 12 months of age	[134]
	*γ-proteobacteria*	*Enterobacteriaceae*	↑ in APP/PS1 mice at 6 months of age	[90]
*Verrucomicrobia*	*Verrucomicrobiae*	*Verrucomicrobiaceae * (*Akkermansia* spp.)	↑↑ in APP/PS1 at 2–12 months of age	[90,111,130]
			N.D. in 5xFAD mice at 9 weeks of age/↑ at 12 months of age	[134,139]

N.D.: not detected; =: no differences; ↓: decrease; ↑: increase; ↑↑: strong increase.

**Table 2:** Alterations of gut composition in prediabetes and T2DM preclinical models.

Phylum	Class	Family	Diabetes mice (db)	Reference
*Actinobacteria*	*Actinobacteria*	*Coriobacteriaceae*	↑ in db/db mice at 8–12–18 weeks of age	[205]
		*Dermabacteraceae* (Genus *Brachybacterium*)	↑ in db/db mice at 8–12–18 weeks of age	[205]
		*Pseudonocardiaceae* (Genus *Pseudonocardia*)	↑ in db/db mice at 8–12–18 weeks of age	[205]
*Bacteroidetes*	*Bacteroidia*	*Muribaculaceae*	↓ in db/db mice by 8 months of age	[209]
		*Rickenellaceae*	↑ Genus *Rikenella* in HFD induced diabetic mice by 14 weeks of age, after 8 weeks of feeding	[206]
			↑ Genus *Rikenella* in db/db mice by 6 months of age ↓Genus *Alistipes* in db/db mice by 20 weeks -8 months of age	[209,210,211]
		*Bacteroidaceae* (Genus *Bacteroides*)	↓ in db/db mice by 20 weeks of age/ ↑ by 6-8 months of age	[204,209,210,211]
			↓ in ob/ob mice by 8 weeks of age	[211]
	*Bacteroidetes*	*Prevotellaceae*	↑ Genus *Paraprevotella* in db/db mice by 6 months of age ↑ Genus *Alloprevotella* in db/db mice by 6 months of age /↓ by 8 months of age	[209,211]
		*Odoribacteraceae*	↑ in HFD induced diabetic mice by 14 weeks of age, after 8 weeks of feeding	[206]
*Firmicutes*	*Bacilli*	*Streptococcaceae* (Genus: *Lactococcus*)	↑ in HFD induced diabetic mice by 14 weeks of age, after 8 weeks of feeding	[206]
		*Enterococcaceae* (Genus *Enterococcus*)	↑ in db/db mice by 6 months of age	[211]
		*Planococcaceae* (Genus *Kurthia*)	↑ in db/db mice by 6 months of age	[211]
		*Lactobacillaceae* (Genus *Lactobacillus*)	↑ in db/db mice by 14 weeks of age	[206]
	*Clostridia*	*Ruminococcaceae*	↑ in db/db by 8 weeks	[205]
			↑ ob/ob mice by 8 weeks of age ↑ Genera *Ruminococcus_1* and *Oscillibacter* in ob/ob mice by 15–17 weeks of age	[203,206,211]
		*Lachnospiraceae*	↑ Genus *Coprococcus* in HFD induced diabetic mice by 14 weeks of age, after 8 weeks of feeding	[206]
			↑ Genera *Lachnospiraceae_NK4A136* and *Agathobacter* in ob/ob mice by 15–17 weeks of age	[203,206]
			↓ in db/db mice by 20 weeks of age	[204]
		*Clostridiaceae* (Genus *Bacteroides*)	↑ in ob/ob mice by 8 weeks of age	[211]
*Proteobacteria*	*α-Proteobacteria*	*Bradyrhizobiaceae*	↑ in db/db mice at 8-12-18 weeks of age	[205]
	*γ-Proteobacteria*	*Enterobacteriaceae* (Genus *Klebsiella*)	↑in db/db mice by 6 months of age	[211]
		*Moraxellaceae*	↑ in ob/ob mice at 8-12-18 weeks of age	[205]
	*δ-Proteobacteria*	*Desulfovibrionaceae* (Genus *Desulfovibrio*)	↑ in db/db mice by 12 weeks	[205]
			↑ in HFD induced diabetic mice by 14 weeks of age, after 8 weeks of feeding	[206]
	*ε-Proteobacteria*	*Helicobacteraceae*	↓ Genus *Helicobacter* in STZ-induced diabetic mice with cognitive disorder	[212]
			↑ in HFD induced diabetic mice by 14 weeks of age, after 8 weeks of feeding	[206]
*Deferribacteres*	*Deferribacteres*	*Deferribacteraceae*	↑ Genera *Deferribacter* and *Mucispirillum* in HFD induced diabetic mice by 14 weeks of age, after 8 weeks of feeding	[206]

↓: decrease; ↑: increase.

**Table 3:** Alterations of gut composition in AD and metabolic disease animal models.

Phylum	Class	Family	Mixed-Model (AD-Metabolic Disease)	Ref.
*Bacteroidetes*	*Bacteroidia*	*Muribaculaceae*	↓ in 3xTg-AD-HFD mice	[106]
	*Bacteroidetes*	*Prevotellaceae*	↓Genus *Prevotella* in E4FAD mice	[248]
*Firmicutes*	*Erysipelotrichia*	*Erysipelotrichaceae*	↑ Genus *Turicibacter* in E4FAD mice	[248]
	*Clostridia*	*Clostridiaceae*	↑↑ Genus *Clostridium* in 3xTg-AD-HFD mice	[106]
		*Dehalobacteriaceae*	↓ in 3xTg-AD-HFD mice	[106]
		*Peptococcaceae*	↓ in 3xTg-AD-HFD mice	[106]
	*Bacilli*	*Lactobacillaceae*	↓ Genus *Lactobacillus* in E4FAD mice	[248]
		*Streptococcaceae*	Genus *Lactococcus* not present in APOE-/- mice	[235]
*Proteobacteria*	*γ-proteobacteria*	*Enterobacteriaceae*	↑ Genus *Escherichia* in E4FAD mice	[248]
*Verrucomicrobia*	*Verrucomicrobiae*	*Verrucomicrobiaceae* (*Akkermansia* spp.)	↑ in E4FAD mice	[248]

↓: decrease; ↑: increase; ↑↑: strong increase.
